# Changes in postoperative infections following implementation of safe surgery checklist and hand hygiene in University of Gondar Hospital

**DOI:** 10.1186/1753-6561-5-S6-P195

**Published:** 2011-06-29

**Authors:** GM Geathun, A Desalegn

**Affiliations:** 1 Surgery, University of Gondar Hospital, University of Gondar, Ethiopia; 2 Obstetrics, University of Gondar, Ethiopia; 3 Surgery, University of Gondar Hospital, Gondar, Ethiopia

## Introduction / objectives

To measure the changes in post opertive wound infections before and after introduction of hand hygiene and safe surgery check list.

To see the compliance of health workers on hand hygiene and on using the safe surgery check list.

## Methods

Baseline assemnet made on wound infection rate of 100 patients before the impelemenation of hand hygiene using the WHO accreditted alcohol based hand rub technique. Six monthe after, surgical site wound infection in 100 pateints of similar profile is reaudited. Wound infection is declared if there are clinical signs of wound infection from day 3 on post operative course. Wounds already infected during or befoe surgery were excluded. Study was conducted in two wards namely general surgical and Obstetric wards.

## Results

Post operative wound infection rate in surgical wards was reduced by 45% while surgical site infection in obstetric wards went down by 33% after health workers used the hand hygiene in and check list in 50% of the cases.

## Conclusion

the implementation of hand hygiene and safe surgery check list was only 50 % while it brought about significant changes surgical site infection in both wards. the impact on paient safety in genreal and surgical site infection rates will go down by fully implementing the hand hygiene and safe srgery check list.

## Disclosure of interest

None declared.

